# Exploring the HIV continuum of care among young black MSM

**DOI:** 10.1371/journal.pone.0179688

**Published:** 2017-06-29

**Authors:** Lisa Hightow-Weidman, Sara LeGrand, Seul Ki Choi, Joseph Egger, Christopher B. Hurt, Kathryn E. Muessig

**Affiliations:** 1 Institute of Global Health and Infectious Diseases, University of North Carolina at Chapel Hill, Chapel Hill, North Carolina, United States of America; 2 Duke Global Health Institute, Duke University, Durham, North Carolina, United States of America; 3 Department of Health Behavior, Gillings School of Public Health, University of North Carolina at Chapel Hill, Chapel Hill, North Carolina, United States of America; National and Kapodistrian University of Athens, GREECE

## Abstract

**Background:**

HIV disproportionately impacts young, black men who have sex with men (YBMSM) who experience disparities across the HIV care continuum. A more nuanced understanding of facilitators and barriers to engagement in care, missed visits, antiretroviral uptake, adherence and viral suppression could improve care and intervention design.

**Methods:**

A randomized controlled trial of an online intervention, healthMpowerment, enrolled 465 YBMSM (18–30 years); 193 identified as HIV-positive. Bivariable and multivariable analyses of baseline data explored predictors of: engagement in care, missed visits, antiretroviral uptake, self-reported adherence, and viral suppression.

**Results:**

Mean age was 24.9 years; most identified as gay (71.0%) and were receiving HIV care (89.1%). Among those in care, 52.1% reported no missed visits in the past 12 months, 41 (24.6%) reported one missed visit, and 39 (23.4%) reported two or more. Having insurance (prevalence odds ratio [POR] 4.5; 95% CI: 1.3, 15.8) and provider self-efficacy (POR 20.1; 95% CI: 6.1, 64.1) were associated with being in care. Those with a college degree (POR 9.1; 95% CI: 1.9, 45.2) and no recent marijuana (POR 2.6; 95% CI: 1.2, 5.6) or methamphetamine use (POR 5.4; 95% CI: 1.0, 28.5) were less likely to miss visits. Most (n = 153, 84.1%) had been prescribed antiretroviral therapy. A majority of participants (70.8%) reported ≥90% adherence; those with depressive symptoms had 4.7 times the odds of reporting adherence <90% (95% CI: 1.65, 13.37). Of participants who reported viral load testing in the past six months, 65% (n = 102) reported an undetectable viral load. Disclosure to sex partners was associated with viral suppression (POR 6.0; 95% CI: 1.6, 22.4).

**Conclusions:**

Multi-level facilitators and barriers to engagement across the continuum of care were identified in this sample of YBMSM. Understanding the distinct needs of YBMSM at each stage of the continuum and addressing them through tailored approaches is critical for long term success in care.

## Background

Black men who have sex with men (BMSM) experience disparities along all stages of the HIV care continuum. Compared with other men who have sex with men (MSM), BMSM are more likely to test positive or have undiagnosed HIV [1, 2]. More BMSM compared to White or Latino MSM are infected-but-unaware [1, 3]. Moreover, HIV-positive BMSM are less likely to be engaged in care, access or adhere to antiretroviral therapy (ART), or be virally suppressed compared with other HIV-positive MSM [1, 3–5]. Data from 3 waves (2008, 2011, 2014) of the National HIV Behavioral Surveillance (NHBS) found that despite improvements in ART coverage among all groups of MSM, BMSM were less likely to be taking ART in each of the 3 study years, even after controlling for education, income and health insurance [6]. While many continuum-based studies have focused on groups who are already engaged in care [7–10], one notable exception is Rosenberg, et al., who used Centers for Disease Control and Prevention (CDC) estimates of infection to construct HIV care continua for Black and White MSM for 2009–2010. Their modeling suggested that only 24% of BMSM were in care and 16% were virally suppressed [11].

There are also disparities in the care continuum related to age. Among those aged 13–24 years diagnosed with HIV in the United States (US), approximately 38% have achieved viral suppression [12]. One recent study conducted within the Adolescent Medicine Trials Network for HIV Interventions (ATN) found that among 1548 adolescents and young adults (81% male, 72% Black, 70% gay/bisexual) initially identified with HIV infection, 68% were linked to care, 54% were engaged in care, 31% started antiretroviral therapy (ART), and 7% reached an undetectable viral load (VL) during the study period. This is substantially lower than the estimated 50% of persons achieving viral suppression across all age groups [12, 13].

While much of the literature has focused on identifying factors that affect HIV rates among young BMSM (YBMSM) [3, 4], the relationship between psychosocial, structural, and social network factors (e.g. social isolation, social support) and HIV care outcomes has been less fully explored. Two recent studies focusing on YBMSM in care reported disparate results. While Hussen, et al., observed no associations between social support, psychological distress and continuum of care outcomes in a study of 132 HIV-positive YBMSM recruited from adolescent medicine HIV clinics [14], Magnus, et al., found that among a sample of newly engaged in care HIV-positive young MSM of color (n = 224, 73% Black), those with a history of depression were less likely to report missed visits [15]. In a population-based sample of 214 YBMSM, a history of criminal justice involvement was found to be positively associated with both retention in care and viral suppression [16]. These prior studies notwithstanding, a greater understanding of how multilevel barriers and facilitators experienced by YBMSM impact the care continuum, from engagement through viral suppression, can provide valuable information to improve provision of care and the design of interventions.”

HealthMpowerment.org (HMP) is a mobile- phone-optimized, Internet-based intervention for both HIV-positive and negative YBMSM (age 18–30) that provides HIV and sexually transmitted infection (STI) prevention and care information, resources, tailored feedback, game-based elements and a social networking platform to offer and receive social support from peers [17, 18]. This paper presents baseline data from HIV positive participants in the HMP study to explore potential sociodemographic, psychosocial, and HIV care-related predictors of outcomes along the HIV care continuum among YBMSM.

## Methods

### Sample

A randomized controlled trial comparing HMP to a control website that provided HIV/STI information enrolled 465 YBMSM between November 2013 and October 2015. Participants completed an online baseline survey at an in-person visit and follow-up assessments were conducted online at 3, 6 and 12-months post enrollment. Baseline data from 193 participants who identified as HIV positive at baseline are presented here. Written informed consent was obtained from participants at the time of study enrollment. This study was approved by the University of North Carolina at Chapel Hill Institutional Review Board.

### Eligibility criteria

Study eligibility criteria at enrollment were: (1) age 18 to 30; (2) born biologically male; (3) self-identify as Black; (4) currently reside in North Carolina; (5) currently have access to a mobile device (e.g. smartphone, tablet) that connects to the internet and has texting capabilities; (6) any of the following in the past six months: (a) condomless anal sex with a male partner, (b) any anal sex with more than three male sex partners, (c) exchange of money, gifts, shelter, or drugs for anal sex with a male partner, or (d) anal sex while under the influence of drugs or alcohol (i.e., high or drunk within two hours of sex).

### Recruitment

Study flyers inviting individuals to participate were posted at local college campuses and previously identified off-campus venues (bars, clubs, coffee shops) frequented by YBMSM. Advertisements and targeted messages were posted on websites, including Craigslist, relevant Facebook groups, and Grindr. Participants were also recruited through word of mouth and flyers at local case management organizations, and HIV/STI clinics.

### Measures

Sociodemographic items assessed age, level of education, income, arrests (last 3 months), homelessness (past 6 months), health insurance, and sexual identity (gay vs. non-gay).

#### Psychosocial

Social support: The Medical Outcomes Study Social Support Survey (MOS-SSS) was used to measure perceived social support [19]. Subscales for emotional/informational (8 items) (alpha = 0.97), tangible (4 items) (alpha = 0.92), affectionate (3 items) (alpha = 0.94), and positive social interaction (3 items) (alpha = 0.96) were calculated. Item responses were based on a 5-point Likert scale. Subscale scores were transformed so that the lowest possible score was 0 and the highest possible score was 100; higher scores indicate greater perceived social support.

Social isolation: Social isolation was derived from the 6-item version of the Lubben Social Network Scale (alpha = 0.83) [20]. The scale assesses social network size and the ease and frequency of contact with network members (range = 0–30) with a higher score indicating a lower likelihood of social isolation. Individuals with a score <12 were considered socially isolated.

Depression: Depressive symptoms were assessed with the Center for Epidemiologic Studies Depression Scale (CES-D), a 20-item validated survey of clinically significant distress as a marker for clinical depression (alpha = 0.90) [21]. A dichotomous depression variable was created using a CES-D score of ≥16, which suggests clinically relevant depressive symptomology.

Anxiety: The seven-item version of the General Anxiety Disorder scale (GAD-7) was used to measure anxiety (alpha = 0.93) [22]. Items measured the frequency with which respondents experienced anxiety symptoms in the past two weeks. Scores of 0, 1, 2, and 3, were assigned to the response categories of “not at all,” “several days,” “more than half the days” and “nearly every day,” respectively (range 0–21). Scores of 5, 10, and 15 represent cut points for mild, moderate, and severe anxiety, respectively.

Substance use: Substance use items assessed any use of alcohol, marijuana or synthetic marijuana, crack or powder cocaine, methamphetamine, club drugs (e.g., ketamine, methylenedioxymethamphetamine [MDMA], gamma-hydroxybutyrate [GHB]), opiates and inhalants in the past 3 months.

HIV disclosure: Participants were asked to indicate how many friends, family and sex partners they had disclosed their HIV status to with response options of none, a few, most, or all. Each disclosure variable was dichotomized to indicate disclosure to none versus a few, most, or all.

#### HIV care related

Self-efficacy for communicating with health care provider: Six items based on a 5-point Likert scale were adapted from Shively et al. [23] to measure communication with HIV care providers among HIV-positive participants (alpha = 0.96). Scores of 1 through 5 were assigned to responses of “not at all sure,” “somewhat sure,” “moderately sure,” “very sure” and “totally sure,” respectively. Mean scores ≥4 were categorized as high self-efficacy for communicating with health care provider, while scores < 4 were categorized as low self-efficacy.

Engagement in care: To assess engagement in HIV care, participants were asked if they were currently enrolled in or receiving HIV care of any kind. Participants were also asked to describe reasons for not being in care.

Missed HIV care appointments: Adherence to scheduled medical appointments with HIV care providers over the past 12 months was assessed by self-reported number of missed visits. We dichotomized missed appointments into no missed HIV medical appointments or having one or more missed appointments [24, 25]. Missed visits are the most immediately available measure of retention in care and are associated with mortality [25].

Antiretroviral (ART) uptake: Participants were asked about current and ever (start date) ART use. Participants who had never used ART were asked to describe their reasons (e.g. don’t need them, no money or insurance, worried about side effects).

Medication adherence/viral suppression: A Visual Analogue Scale was used to assess medication adherence. Participants selected a value ranging from 0–100% to indicate their adherence on a regular basis [26]. A dichotomous variable was created with values greater than or equal to 90% classified as adherent [27]. Viral suppression was provided via self-report for those who reported having had a viral load performed in the past 6 months using a two-part question: 1) was your viral load undetectable and 2) If not undetectable, participants were asked to indicate the range that most closely matched their last viral load (e.g. 20–500 copies, 501–9,999 copies).

### Statistical analysis

Logistic regression models of baseline data were used to explore sociodemographic, psychosocial, and HIV care-related predictors of five key outcomes: engagement in care, no missed visits, ART uptake, self-reported adherence and viral suppression. Independent predictors of these outcomes were selected based on empirical considerations established through formative work and a thorough review of the literature. The final selection of variables chosen were those found to impact both HIV acquisition risk and engagement in the HIV continuum of care for both YBMSM, our target population, as well as other HIV infected populations. Prevalence odds ratios (POR) with 95% confidence intervals were calculated as the measure of association. Bivariate logistic regression was conducted with each independent variable for all five outcome variables and multivariable logistic regression models with stepwise backward selection constructed for each outcome variable. All independent factors significant at the p≤0.1 level in bivariable analyses were included in multivariable logistic regression analysis and predictors with the highest p-value were deleted from the model in a step-wise fashion. The model with lower Akaike Information Criterion (AIC) values was considered the better model. For significant bivariable factors that were highly correlated with one another (i.e., collinear, r ≥ 0.70), the factor with the lowest AIC in the model was retained in the final multivariable model. No alpha adjustment for multiple testing was applied. All analyses were performed using SAS 9.4 (SAS Institute, Cary, NC).

## Results

### Description of sample

Mean age was 24.9 years; most identified as gay (71.0%) (Table 1). Forty percent of the sample was socially isolated and 112 (58.6%) received a CES-D score that met criteria for clinical depression. About one-third (33.2%) of the sample had a history of homelessness in the prior six months. Substance use was common with 79.3% reporting alcohol use, 68.4% reporting marijuana use, 14.0% crack/cocaine use and 7.3% methamphetamine use.

**Table 1:** Descriptive statistics of HIV-positive HMP participants at study entry.

		(n = 193)

		N (%)
		Mean [SD]
Age (n = 192)		24.91 [3.11]
Education		
	• < High school	21 (10.9)
	• High school/GED, Some technical/college	138 (71.5)
	• College degree or more	34 (17.6)
Income (n = 191)		
	• < $10,999	102 (53.4)
	• $11,000-$20,999	41 (21.5)
	• $21,000–30,999	26 (13.6)
	• >/ = $31,000	22 (11.5)
Arrested (last 3 months)		13 (6.7)
Homeless (last 6 months)		64 (33.2)
Health insurance		155 (80.3)
How do you best describe yourself?		
	• Gay	138 (71.5)
	• Non-gay	55 (28.5)
Social support		
	• Emotional/Informational (n = 191)	69.9 [29.2]
	• Tangible (n = 191)	65.2 [31.0]
	• Affectionate (n = 189)	68.7 [32.6]
	• Positive social interaction (n = 191)	68.5 [30.6]
Social isolation (n = 192)		77 (40.1)
Depression (n = 191)		112 (58.6)
Anxiety (n = 189)		
	• Minimal/Mild	130 (68.8)
	• Moderate/Severe	59 (31.2)
Substance use (past 3 months)		
	• Alcohol	153 (79.3)
	• Marijuana	132 (68.4)
	• Crack and powder cocaine	27 (14.0)
	• Methamphetamine	14 (7.3)
	• Club drugs (Ecstasy, Ketamine, GHB)	14 (7.3)
	• Opiates	10 (5.2)
	• Inhalants	14 (7.3)
Disclosure of HIV status to **friends** (n = 168)		
	• None	25 (14.9)
	• A few/Most people/All	143 (85.1)
Disclosure of HIV status to **family** (n = 168)		
	• None	39 (23.2)
	• A few/Most people/All	129 (76.8)
Disclosure of HIV status to **sex partners** (n = 168)		
	• None	15 (8.9)
	• A few/Most people/All	153 (91.1)
Self-efficacy for health care provider (HCP) communication		
	• Mean <4	47 (24.4)
	• Mean ≥4	146 (80.2)
Engagement in care		172 (89.1)
No missed HIV care visits (n = 167)		87 (52.1)
Antiretroviral uptake (n = 182)		153 (84.1)
Antiretroviral adherence (VAS) (> = 90) (n = 137)		97 (70.8)
Virally suppressed (n = 157)		102 (65.0)

### Engagement in care

Most participants, 172 (89.1%) reported being currently enrolled in or receiving HIV care. The most common reasons for not being in care were inconvenient location or transportation issues (n = 9, 42.9%) and cost concerns (n = 7, 33.3%). A majority of participants (n = 146. 80.2%) reported a high degree of self-efficacy for communicating with their health care provider. In multivariable analyses (Table 2), having insurance (POR 8.47; 95% CI: 2.59, 27.73) and having high self-efficacy for communication with a health care provider (POR 13.19; 95% CI: 3.72, 46.77) were associated with being in care.

**Table 2:** Bivariable and multivariable analysis for engagement in care and no missed visits.

		Engagement in Care^a^				No Missed Visits^b^			
		Unadjusted POR (95% CI)	p-value	Adjusted POR (95% CI)	p-value	Unadjusted POR (95% CI)	p-value	Adjusted POR (95% CI)	p-value
Age		0.99 (0.85, 1.15)	0.89	—	—	0.93 (0.85, 1.03)	0.17	—	—
Education									
	• < High School	1.00	0.59			1.00	**0.01**	1.00	**0.02**
	• High school/GED, Some technical/college	1.30 (0.42, 6.18)	0.49	—	—	3.42 (1.05, 11.07)	0.04	3.62 (1.00, 13.11)	0.70
	• >/ = College degree	0.97 (0.21, 4.54)	0.97			8.53 (2.14, 34. 09)	**<0.01**	9.43 (1.91, 46.66)	**0.01**
Income							**0.10**		0.12
	• < $10,999	1.00	0.83			1.00		1.00	
	• $11,000-$20,999	1.01 (0.30, 3.41)	0.99	—	—	1.81 (0.81, 4.04)	0.15	1.03 (0.41, 2.59)	0.29
	• $21,000–30,999	0.60 (0.17, 2.09)	0.42			0.61 (0.23, 1.60)	0.32	0.27 (0.08, 0.85)	0.02
	• >/ = $31,000	0.69 (0.17, 2.74)	0.60			2.32 (0.81, 6.64)	0.12	0.95 (0.27, 3.27)	0.53
Arrested (last 3 months)									
	• Yes	1.00		—	—	1.00		—	—
	• No	0.67 (0.08, 5.40)	0.70			0.60 (0.17, 2.14)	0.43		
Homeless (last 6 months)									
	• Yes	1.00		1.00		1.00		1.00	
	• No	2.47 (0.99, 6.17)	**0.05**	1.02 (0.32, 3.25)	0.98	2.12 (1.08, 4.15)	**0.03**	1.58 (0.70, 3.56)	0.27
Health insurance									
	• Yes	9.55 (3.60, 25.39)	**<0.01**	8.47 (2.59, 27.73)	**<0.01**	0.61 (0.25, 1.48)	0.27	—	—
	• No	1.00		1.00		1.00			
How do you best describe yourself?				—	—			—	—
	• Non-gay	1.00				1.00			
	• Gay	1.29 (0.49, 3.40)	0.60			1.51 (0.77, 2.98)	0.23		
Social support									
	• Emotional/Informational^c^	1.21 (1.04,1.40)	**0.01**			1.12 (1.00, 1.25)	**0.04**	1.09 (0.95, 1.26)	0.24
	• Tangible^c^	1.23 (1.07, 1.42)	**<0.01**	1.16 (0.97, 1.39)	0.10	1.12 (1.00, 1.24)	**0.05**		
	• Affectionate^c^	1.08 (0.95, 1.23)	0.26			1.01 (0.91, 1.11)	0.87		
	• Positive Social Interaction^c^	1.04 (0.90, 1.20)	0.64			1.01 (0.91, 1.12)	0.88		
Social isolation									
	• Yes	1.00		—	—	1.00		1.00	
	• No	1.96 (0.77, 4.99)	0.16			2.13 (1.13, 4.04)	**0.02**	1.26 (0.54, 2.94)	
Depression									
	• Yes	1.00		—	—	1.00		—	—
	• No	1.47 (0.56, 3.83)	0.43			1.48 (0.80, 2.76)	0.22		
Anxiety									
	• Minimal/Mild	1.42 (0.55, 3.62)	0.47	—	—	0.90 (0.46, 1.75)		—	—
	• Moderate/Severe	1.00				1.00	0.75		
Alcohol									
	• Yes	1.00		—	—	1.00		—	—
	• No	1.13 (0.36, 3.55)	0.84			1.04 (0.49, 2.22)	0.91		
Marijuana									
	• Yes	1.00		—	—	1.00		1.00	
	• No	1.56 (0.54, 4.43)	0.42			2.62 (1.32, 5.19)	**0.01**	2.49 (1.14, 5.44)	**0.02**
Crack/powder cocaine									
	• Yes	1.00		—	—	1.00		—	—
	• No	1.52 (0.47, 4.93)	0.48			1.68 (0.68, 4.18)	0.26		
Methamphetamine									
	• Yes	1.00		—	—	1.00		1.00	
	• No	2.44 (0.62, 9.57)	0.20			4.72 (0.97, 22.95)	**0.05**	5.42 (1.03, 28.52)	**0.05**
Club drugs									
	• Yes	1.00		—	—	1.00		—	—
	• No	0.71 (0.15, 3.42)	0.67			1.11 (0.33, 3.79)	0.87		
Opiates									
	• Yes	1.00		—	—	1.00		—	—
	• No	2.16 (0.43,10.91)	0.35			0.42 (0.08, 2.23)	0.31		
Inhalants									
	• Yes	1.00		—	—	1.00		—	—
	• No	2.44 (0.62, 9.57)	0.20			3.11 (0.80, 12.17)	**0.10**		
Disclosure of HIV status to **friends**				—	—			—	—
	• None	1.00				1.00			
	• A few/ Most people/All	0.62 (0.08, 5.12)	0.66			1.61 (0.66, 3.95)	0.29		
Disclosure of HIV status to **family**				—	—			—	—
	• None	1.00				1.00			
	• A few/ Most people/All	1.45 (0.36, 5.91)	0.60			1.20 (0.56, 2.58)	0.63		
Disclosure of HIV status to **sex partners**				—	—			—	—
	• None	1.00				1.00			
	• A few/ Most people/All	1.14 (0.14, 9.69)	0.90			0.81 (0.25, 2.68)	0.73		
Self-efficacy for HCP communication									
	• Mean <4	1.00		1.00		1.00		—	—
	• Mean ≥4	20.12 (6.12, 64.05)	**<0.01**	13.19 (3.72, 46.77)	**<0.01**	0.66 (0.29, 1.50)	0.32		

a 193 were included in bivariable analysis; 191 were included multivariable analysis due to missing independent variable values. Emotional/Informational social support and tangible social support were significant in bivariable analysis, but tangible social support was included in the multivariable model with lower AIC.

b 167 were included in bivariable analysis; 162 were included multivariable analysis due to missing independent variable values. Emotional/Informational social support and tangible social support were significant in bivariable analysis, but emotional/informational social support was included in the multivariable model with lower AIC.

c The odds for 10 units increase in social support.

### No missed HIV care visits

Among those in care, 87 (52.1%) reported no missed visits in the past 12 months, 41 (24.6%) reported one missed visit, and 39 (23.4%) reported two or more. Of those reporting missed visits (n = 80), most had missed visits in the past 3 months (70.0%). In multivariable analysis, those with a college degree (POR 9.43; 95% CI: 1.91, 46.66) and those with no marijuana use (POR 2.49; 95% CI: 1.14, 5.44) and no methamphetamine use in the prior 3 months (POR 5.42, 95% CI: 1.03, 28.52) were less likely to miss visits.

### Antiretroviral uptake

Eighty-four percent were on ART (n = 153). Among the 29 YBMSM not currently taking ART, the most common reasons were participants’ concerns regarding their ability to adhere to medication (34.5%), fear of side effects (24.1%) and not having found a doctor (27.6%). The mean time from diagnosis to ART uptake was 9.4 months; the median was 2.4 months. In multivariable analysis (Table 3), having insurance (POR 6.74; 95% CI: 2.43, 18.74) and not using cocaine or crack (POR 5.87; 95% CI: 1.60, 21.48) were associated with ART uptake.

**Table 3:** Bivariable and multivariable analysis for antiretroviral therapy uptake.

		ART Uptake^a^			
		Unadjusted POR (95% CI)	p-value	Adjusted POR (95% CI)	p-value
Age		1.05 (0.92, 1.19)	0.47	—	—
Education					
	• < High School	1.00	0.69		
	• High school/GED, Some technical/college	1.57 (0.47, 5.25)	0.46	—	—
	• >/ = College degree	1.16 (0.28, 4.76)	0.84		
Income					
	• < $10,999	1.00	0.40		
	• $11,000-$20,999	0.60 (0.24, 1.52)	0.28	—	—
	• $21,000–30,999	0.86 (0.26, 2.88)	0.81		
	• >/ = $31,000	3.43 (0.43, 27.62)	0.25		
Arrested (last 3 months)					
	• Yes	1.00		1.00	
	• No	3.78 (1.14, 12.51)	**0.03**	1.65 (0.35, 7.87)	0.53
Homeless (last 6 months)					
	• Yes	1.00		1.00	
	• No	2.57 (1.15, 5.76)	**0.02**	1.53 (0.57, 4.15)	0.40
Health insurance					
	• Yes	7.00 (2.91, 16.86)	**<0.01**	6.74 (2.43, 18.74)	**<0.01**
	• No	1.00		1.00	
How do you best describe yourself?				—	—
	• Non-gay	1.00			
	• Gay	1.39 (0.60, 3.24)	0.44		
Social support					
	• Emotional/Informational^b^	1.08 (0.95, 1.24)	0.25		
	• Tangible^b^	1.12 (0.98, 1.27)	**0.09**	1.04 (0.89, 1.22)	0.64
	• Affectionate^b^	1.08 (0.95, 1.22)	0.23		
	• Positive Social Interaction^b^	1.04 (0.91, 1.19)	0.54		
Social isolation					
	• Yes	1.00		—	—
	• No	0.96 (0.43, 2.18)	0.93		
Depression					
	• Yes	1.00		—	—
	• No	1.27 (0.55, 2.94)	0.57		
Anxiety					
	• Minimal/Mild	1.57 (0.68, 3.64)	0.29	—	—
	• Moderate/Severe	1.00			
Alcohol					
	• Yes	1.00		—	—
	• No	0.77 (0.30, 1.96)	0.58		
Marijuana					
	• Yes	1.00		—	—
	• No	1.53 (0.61, 3.81)	0.37		
Crack/powder cocaine					
	• Yes	1.00		1.00	
	• No	7.01 (2.79, 17.60)	**<0.01**	5.87 (1.60, 21.48)	**<0.01**
Methamphetamine					
	• Yes	1.00		—	—
	• No	2.56 (0.73, 8.95)	0.14		
Club drugs					
	• Yes	1.00		1.00	
	• No	0.35 (0.10, 1.23)	**0.10**	0.92 (0.10, 8.61)	0.94
Opiates					
	• Yes	1.00		1.00	
	• No	4.74 (1.19, 18.85)	**0.03**	2.35 (0.22, 25.44)	0.48
Inhalants					
	• Yes	1.00		1.00	
	• No	3.78 (1.14, 12.51)	**0.03**	1.35 (0.28, 6.53)	0.71
Disclosure of HIV status to **friends**				—	—
	• None	1.00			
	• A few/ Most people/All	0.20 (0.03, 1.52)	0.12		
Disclosure of HIV status to **family**				—	—
	• None	1.00			
	• A few/ Most people/All	1.98 (0.80, 4.88)	0.14		
Disclosure of HIV status to **sex partners**				—	—
	• None	1.00			
	• A few/ Most people/All	0.83 (0.18, 3.90)	0.81		
Self-efficacy for HCP communication					
	• Mean <4	1.00		1.00	
	• Mean ≥4	3.13 (1.32, 7.42)	**0.01**	1.98 (0.63, 6.16)	0.24

a 182 were included in bivariable analysis; 180 were included multivariable analysis due to missing independent variable values.

b Odds for 10 unit increase in social support.

### Self-reported adherence and viral suppression

A majority of participants (70.8%) reported ≥90% adherence. In bivariable analyses (Table 4), lack of depressive symptoms, higher levels of tangible support, and not being homeless in the past 6 months were associated with self-reported adherence ≥90%. In multivariable analyses, those with depressive symptoms had 4.7 times the odds of reporting adherence <90% (95% CI: 1.65, 13.37).

**Table 4:** Bivariable and multivariable analysis for self-reported antiretroviral adherence and viral suppression.

		Self-Reported Adherence (≥90)^a^				Self-Reported Viral Suppression^b^			
		Unadjusted POR (95% CI)	p-value	Adjusted POR (95% CI)	p-value	Unadjusted POR (95% CI)	p-value	Adjusted POR (95% CI)	p-value
Age		0.94 (0.83, 1.06)	0.30	—	—	1.05 (0.95, 1.17)	0.34	—	—
Education									
	• < High School	1.00	0.25			1.00	**0.04**	1.00	0.19
	• High school/GED, Some technical/college	2.22 (0.74, 6.70)	0.16	—	—	4.02 (1.38, 11.73)	**0.01**	3.32 (0.91, 12.13)	0.12
	• >/ = College degree	3.15 (0.76, 13.00)	0.11			3.90 (1.06, 14.33)	**0.04**	2.60 (0.57, 11.80)	0.54
Income									
	• < $10,999	1.00	0.29			1.00	0.21		
	• $11,000-$20,999	1.91 (0.69, 5.32)	0.22	—	—	1.68 (0.71, 3.97)	0.24	—	—
	• $21,000–30,999	0.87 (0.28, 2.67)	0.81			1.46 (0.53, 3.99)	0.46		
	• >/ = $31,000	2.78 (0.74, 10.45)	0.13			3.65 (0.98, 13.56)	0.05		
Arrested (last 3 months)									
	• Yes	1.00		—	—	1.00		—	—
	• No	0.97 (0.18, 5.21)	0.97			1.60 (0.47, 5.50)	0.46		
Homeless (last 6 months)									
	• Yes	1.00		1.00		1.00		1.00	
	• No	3.41 (1.56, 7.44)	**<0.01**	2.04 (0.86, 4.85)	0.11	2.61 (1.28, 5.34)	**0.01**	2.26 (0.98, 5.21)	0.06
Health insurance									
	• Yes	1.66 (0.59, 4.64)		—	—	1.28 (0.49, 3.34)		—	—
	• No	1.00	0.34			1.00	0.62		
How do you best describe yourself?				—	—			—	—
	• Non-gay	1.00				1.00			
	• Gay	1.73 (0.78, 3.86)	0.18			1.50 (0.73, 3.08)	0.27		
Social support									
	• Emotional/Informational^c^	1.12 (0.99, 1.27)	**0.07**	0.97 (0.82, 1.15)	0.74	1.01 (0.90, 1.13)	0.87		
	• Tangible^c^	1.14 (1.01, 1.29)	**0.04**			0.99 (0.88, 1.10)	0.79	—	—
	• Affectionate^c^	1.04 (0.92, 1.16)	0.54			0.97 (0.87, 1.08)	0.55		
	• Positive Social Interaction^c^	1.09 (0.97, 1.23)	0.17			0.97 (0.87, 1.08)	0.60		
Social isolation									
	• Yes	1.00		1.00		1.00		—	—
	• No	2.04 (0.96, 4.35)	**0.06**	1.16 (0.42, 3.22)	0.78	0.70 (0.35, 1.38)	0.30		
Depression									
	• Yes	1.00		1.00		1.00		—	—
	• No	4.91 (1.98, 12.19)	**<0.01**	4.70 (1.65, 13.37)	**<0.01**	1.15 (0.59, 2.24)	0.68		
Anxiety									
	• Minimal/Mild	1.29 (0.59, 2.83)	0.53	—	—	0.73 (0.35, 1.56)	0.42	—	—
	• Moderate/Severe	1.00				1.00			
Alcohol									
	• Yes	1.00		—	—	1.00		—	—
	• No	0.48 (0.20, 1.17)	0.11			1.10 (0.49, 2.48)	0.82		
Marijuana									
	• Yes	1.00		—	—	1.00		—	—
	• No	1.78 (0.76, 4.17)	0.19			1.33 (0.66, 2.67)	0.43		
Crack/powder cocaine									
	• Yes	1.00		—	—	1.00		1.00	
	• No	1.09 (0.31, 3.75)	0.90			3.28 (1.25, 8.60)	**0.02**	2.59 (0.84, 8.00)	0.10
Methamphetamine									
	• Yes	1.00		—	—	1.00		1.00	
	• No	1.23 (0.29, 5.18)	0.78			4.04 (0.97, 16.84)	**0.06**	3.61 (0.64, 20.37)	0.15
Club drugs									
	• Yes	1.00		—	—	1.00		1.00	
	• No	1.03 (0.19, 5.56)	0.97			0.33 (0.09, 1.24)	**0.10**	1.68 (0.35, 8.00)	0.52
Opiates									
	• Yes	1.00		—	—	1.00		—	—
	• No	1.65 (0.27, 10.27)	0.59			2.59 (0.56, 12.00)	0.23		
Inhalants									
	• Yes	1.00		—	—	1.00		—	—
	• No	0.80 (0.15, 4.13)	0.79			2.45 (0.63, 9.53)	0.20		
Disclosure of HIV status to **friends**									
	• None	1.00		—	—	1.00		—	—
	• A few/ Most people/All	1.83 (0.71, 4.72)	0.21			0.57 (0.20, 1.66)	0.30		
Disclosure of HIV status to **family**									
	• None	1.00		—	—	1.00		—	—
	• A few/ Most people/All	0.71 (0.26, 1.94)	0.51			0.86 (0.37, 2.00)	0.73		
Disclosure of HIV status to **sex partners**									
	• None	1.00		—	—	1.00		1.00	
	• A few/ Most people/All	1.30 (0.37, 4.61)	0.69			5.18(1.51, 17.79)	**0.01**	6.03 (1.62, 22.40)	**0.01**
Self-efficacy for HCP communication									
	• Mean <4	1.00		1.00		1.00		—	—
	• Mean ≥4	2.25 (0.92, 5.51)	**0.08**	1.24 (0.46, 3.32)	0.68	1.05 (0.43, 2.57)	0.91		

a 137 were included in bivariable analysis; 134 were included multivariable analysis due to missing independent variable values. Emotional/Informational social support and tangible social support were significant in bivariate analysis, but emotional/informational social support was included in the multivariable model with lower AIC.

b 157 were included in bivariable analysis; 145 were included multivariable analysis due to missing independent variable values.

c Odds for 10 unit increase in social support.

Sixty-five percent of HIV-positive YBMSM who reported completing viral load testing in the past six months reported an undetectable viral load (n = 102). In multivariable analyses, any disclosure to sex partners was associated with higher likelihood of viral suppression (POR 6.03; 95% CI: 1.62, 22.40). Not reporting homelessness in the past 6 months approached statistical significance (p = 0.06).

## Discussion

This analysis revealed a range of factors and characteristics associated with suboptimal outcomes for YBMSM on five indicators along the HIV care continuum. Notably, some associations—such as the beneficial effect of health insurance and the detrimental effect of drug use—were significant across more than one continuum outcome. Yet, overall, each outcome had a unique set of associated factors. Understanding these distinct needs of YBMSM at each stage of the continuum and addressing them through tailored approaches is critical for achieving the prevention benefits of viral suppression through “treatment as prevention” and sustaining long-term optimal health outcomes. Below we discuss our findings at each step of the continuum and potential intervention implications for YBMSM.

The majority of HIV-positive men in this study were engaged in care. This is likely related to the fact that among those who were HIV-infected, about half (n = 102) were recruited through case management organizations or HIV providers. Although a potential source of bias, this also highlights the importance of community and health-care related partnerships with research groups to enroll HIV-infected YBMSM in large studies. Further, participants in this study reported a high overall self-efficacy for communicating with their health care providers. While not directly measured in this study, perception of racism and medical mistrust by Black patients, including BMSM, has been found to impact patient-provider communication, health care access, and uptake of prevention services [28, 29]. In prior studies, BMSM were found to be less likely to report primary care provider awareness of their sexual orientation compared to White MSM [30]. However, few studies have examined the relationships between BMSM and primary care providers, particularly among those who are HIV-positive. Additional in-depth research is needed to elucidate barriers and facilitators to optimal and productive patient-provider communication that can be targeted for interventions to impact engagement in care.

Despite high levels of care engagement there were also high rates of missed visits. Missed visits have multiple negative consequences: interruptions in ART due to lapses in insurance or drug assistance coverage, delays in health care (e.g. sexually transmitted infection screening and immunizations), and timely evaluations for ART toxicity and adherence. In this study, missed visits were associated with lower education. These results align with findings from a linkage-to-care study of 1891 adolescents and young adults living with HIV (27.8% perinatally infected; 72.2% behaviorally infected) which found that missed appointments were common among those with less education living in economically disadvantaged areas [31]. While not measured in this study, Harper et al. found that in a sample of 200 HIV-positive male adolescents, 66% of whom were Black, those with a stronger connection to their racial/ethnic identity were less likely to have missed an HIV medical appointment during the prior 3 months, while those with more negative attitudes towards gay/bisexual people were more likely to have missed appointments [32]. These findings thus highlight the importance of addressing individual, developmental and structural factors when possible.

Missed visits among YBMSM in this sample were also associated with marijuana use. The impact of marijuana on continuum of care metrics has been less studied than other substances [33, 34] and the limited studies have reported that the effect of marijuana on retention in care are inconclusive. In a study of 212 HIV seropositive YBMSM in Chicago, there was no significant association found between intermittent or heavy marijuana use and retention in care [35]. Another study, however, found weekly or daily marijuana use to be associated with loss to follow-up [36]. Given the high proportion of marijuana use among people living with HIV [37–41] as well as increasing trends towards legalization of marijuana, additional research specifically addressing the context and frequency of marijuana use and its impact on HIV care and treatment is warranted.

Most participants in this sample were on ART and reported adhering to ≥90% of their prescribed medication. While health insurance and not using cocaine (including crack) were positively associated with ART uptake, depressive symptomology was the sole factor associated with poor adherence. More than half of the sample (59.4%) reported clinically relevant depressive symptoms. It is clear that depression negatively impacts adherence; a meta-analysis across nearly 100 independent samples found that depression was significantly associated with nonadherence (p<0.0001) [42]. Addressing mental health must be an element of care provision across the continuum and in the development of interventions for those YBMSM living with HIV.

Despite overall high levels of engagement in care, ART uptake, and self-reported medication adherence, it is concerning that only 65% of HIV-positive YBMSM in this sample reported an undetectable viral load in the past six months. The finding that disclosure was associated with viral suppression may be interpreted in at least two ways. Disclosure is important for accessing support, which can impact engagement in care, medication adherence and ultimately, viral suppression [43]. Given the cross-sectional nature of this analysis, it is also plausible that those who achieved viral suppression were more likely to disclose their status to an intimate partner. Importantly, both interpretations point toward a need for greater support for status disclosure. Currently, there is a lack of interventions targeting disclosure to sex partners. In a recent review, Conserve et al. identified five trials, of which only three were efficacious and all were delivered in multiple small group sessions limiting widespread implementation [44].

Several cross-cutting factors stand out across the care continuum for YBMSM in this sample. First, substance use among HIV-infected YMSM creates challenges in health care provision and has been previously associated with decreased engagement in care and poor adherence to care and medications [45–47]. Substance use can lead to inaccurate perceptions of time, the inability to adhere to routines, and impaired decision making, all important barriers to retention in care [48]. Future studies should examine both the patterns of use as well as the pathways in which both frequent and infrequent use impact care seeking and adherence behaviors. Co-location or integration of HIV care with substance use treatment to improve long-term health outcomes for YMSM should be considered to address these issues.

Second, a majority of the sample was insured (80.3%). This aligns with data from the NHBS that found that between 2008 and 2014, current insurance status among HIV-positive MSM increased from 75% to 86% (6). Many of the participants in the current study may have considered Ryan White and ADAP (AIDS Drug Assistance Program) benefits as insurance. Another explanation for the high percentage insured given the young age of the sample is the ability to be maintained on parents’ insurance plans. It should also be noted that the Patient Protection and Affordable Care Act was signed into law on March 23rd, 2010, and implementation began in late 2013 –coinciding with HMP enrollment (2013–2015). Indeed, the overall percentage of U.S. adults who were uninsured dropped from 18% in the third quarter of 2013 to 11% in the first quarter of 2016 [49]. Given that having insurance was associated with greater engagement in care and ART uptake in this sample, continued efforts to ensure HIV-positive young men have access to insurance or provision of ART benefits through other means is critical.

This exploratory study has some important limitations. Study outcomes were not rare (i.e., <10%) so odds and odds ratios should not be interpreted as risks. The data included in these analyses are from the baseline survey for an online intervention and are thus cross-sectional in nature, limiting our ability to determine causality. Our engagement and viral suppression measures were assessed via self-report, thus introducing the possibility of both recall and reporting bias [50]. However, all our surveys were conducted online which has been shown to be a reliable and valid method for collecting sensitive information among young adults [51, 52]. Finally, as mentioned above, many of the HIV-positive participants in the intervention were recruited from HIV clinics and community based organizations and thus may not be reflective of the population of those YBMSM who are out of care.

Findings from this study have implications for both future research as well as intervention development for HIV-positive YBMSM. The high rates of depression, social isolation and substance use behaviors seen in this study have been found in other work focused on YBMSM, including those who are HIV-positive [53–55]. These psychosocial conditions, particularly in combination are likely to have an additive impact on continuum of care outcomes for YBMSM. While prior work has established the impact of syndemics on heightened risk for HIV infection among BMSM [55–58], longitudinal studies that not only address the combined impact of syndemics over time, but also provide guidance on how to effectively intervene as early as possible in these men’s lives are needed. Validated clinical screening tools for mental health [59, 60] and substance use disorders [61, 62] should be administered upon initial care engagement and at regular intervals to identify those YBMSM in need of additional services [63]. Given the high reported rates of social isolation experienced by YBMSM in this study, network based interventions, such as Project nGage, which focuses on helping YBMSM identify supportive relationships as a mechanism to promote retention in care, could be beneficial [64].

## Conclusion

The results from this large sample of YBMSM revealed multi-level barriers to engagement across the continuum of care. To our knowledge, this is the largest sample of YBMSM in which each stage of the care continuum was separately addressed. Structural barriers such as insurance status and homelessness as well individual- level behaviors and psychosocial factors such as drug use and depression limited engagement in care and virologic suppression. However, a focus on barriers alone is insufficient to understand and address the complex process of engagement in care and ultimately sustained virologic suppression for YBMSM.

## Supporting information

S1 File COC paper data. (XLSX)
